# Restricted access to antiretroviral treatment for undocumented migrants: a bottle neck to control the HIV epidemic in the EU/EEA

**DOI:** 10.1186/s12889-015-2571-y

**Published:** 2015-12-10

**Authors:** Jessika Deblonde, André Sasse, Julia Del Amo, Fiona Burns, Valerie Delpech, Susan Cowan, Michele Levoy, Lilana Keith, Anastasia Pharris, Andrew Amato-Gauci, Teymur Noori

**Affiliations:** Scientific Institute of Public Health, Epidemiology of Infectious Diseases, Juliette Wytsmanstraat 14, 1050 Brussels, Belgium; Institute of Health Carlos III, National Center for Epidemiology, C/Sinesio Delgado 6, 28029 Madrid, Spain; University College London, Research Department of Infection & Population Health, London, WC1E 6JB UK; Royal Free London NHS Foundation Trust, Pond Street, London, NW3 2QG UK; Public Health England, PHIV & STI Department, 61 Colindale Avenue, London, NW9 5EQ UK; Statens Serum Institut, Department of Infectious Medicine Epidemiology, Artillerivej 5, 2300 Copenhagen S, Denmark; PICUM- Platform for International Cooperation on Undocumented Migrants, Rue du Congrès 37-41 / 5, Brussels, 1000 Belgium; European Centre for Disease Prevention and Control, Surveillance and Response Support Unit, Tomtebodavagen 11A, 171 83 Stockholm, Sweden; European Centre for Disease Prevention and Control, Office of the Chief Scientist, Tomtebodavagen 11A, 171 83 Stockholm, Sweden

**Keywords:** HIV infection, Migrants, Europe, Antiretroviral treatment, Access to health care

## Abstract

**Background:**

In the European Union/European Economic Area (EU/EEA), migrants from high-endemic countries are disproportionately affected by HIV. Between 2007 and 2012, migrants represented 39 % of reported HIV cases. There is growing evidence that a significant proportion of HIV acquisition among migrant populations occurs after their arrival in Europe.

**Discussion:**

Migrants are confronted with multiple risk factors that shape patterns of population HIV susceptibility and vulnerability, which simultaneously affect HIV transmission. Undocumented migrants incur additional risks for contracting HIV due to limited access to adequate health care services, protection and justice, alongside insecure housing and employment conditions.

All EU/EEA countries have ratified a number of international and regional human rights instruments that enshrine access to health care as a human right that should be available to everyone without discrimination.

From a clinical and public health perspective, early HIV care and treatment is associated with viral suppression, improved health outcomes and reductions in transmission risks. A current challenge of the HIV epidemic is to reach the highest proportion of overall viral suppression among people living with HIV in order to impact on HIV transmission.

Although the majority of EU/EEA countries regard migrants as an important sub-population for their national responses to HIV, and despite the overwhelming evidence of the individual and public health benefits associated with HIV care and treatment, a significant number of EU/EEA countries do not provide antiretroviral treatment to undocumented migrants.

**Summary:**

HIV transmission dynamics in migrant populations depend on the respective weight of all risk and vulnerability factors to which they are exposed, which act together in a synergistic way. People who are not linked to HIV care will continue to unwillingly contribute to the on-going transmission of HIV. Following the recommendations of the European Union Agency for Fundamental Rights, ensuring access to HIV-care for all sub-populations, including undocumented migrants, would fulfil the human rights of those populations and also strengthen the control of HIV incidence among those not currently able to access HIV care.

## Background

### Migration in the EU/EEA

Migration flows to and within Europe have shaped societies for thousands of years. Many European Union/European Economic Area (EU/EEA) countries have had longstanding and stable migration patterns with countries outside Europe, whilst others have become countries of residence for more diverse groups of migrants in recent years. There is no universally agreed definition of the term ‘migrant’. The United Nations define a long-term migrant as “a person who moves to a country other than that of his or her usual residence for a period of at least 12 months, so that the country of destination effectively becomes his or her new country of usual residence”. In Europe, migrants are often classified according to characteristics such as region of origin or country of birth, nationality, citizenship, and/or residence status. Variations in data collected and factors used to identify migrant cases present challenges in comparing these groups.

In 2012 there were an estimated 50.8 million foreign-born residents in the 27 countries of the EU. Of these, 33.5 million were born outside the EU and 17.3 million were born in another EU country. An additional 442,000 migrants were reported to be living in the EEA countries Iceland, Norway and Liechtenstein in 2012 [1]. The number of people born abroad includes people that have naturalised and become citizens of the country of residence. In 2012, 67 % (34.1 million) of all foreign-born residents were registered as EU citizens. The majority, 20.4 million, originating from non-EU countries, while the remaining 13.7 million were from within the EU [1].

The terms ‘irregular’, ‘undocumented’ and ‘unauthorized migration’ are used to describe the multi-faceted phenomenon of cross-border movement that takes place outside the regulatory norms of countries of origin, transit and destination. Irregular or undocumented migrants are people “who lack regular residence status in a transit or destination country owing to irregular entry, the expiring of visa, the rejection of an asylum application or other reasons” [2].

Estimates regarding people whose residence status is not currently in line with national regulations of entry, stay or employment inevitably vary. Analysis of available data yielded an estimate that between 1.9 and 3.8 million irregular foreign residents were living in the 27 countries of the EU in 2008, constituting 0.4–0.8 % of the total EU population and 7–13 % of the total foreign population. It was found that irregular entry was the least common pathway into irregular status in the EU; the most common being withdrawal or loss of status and rejection of an application for international protection [3].

In the further development of this debate, we plead for ensuring access to HIV-care for all sub-populations, including undocumented migrants.

### Epidemiological data on HIV among migrants in EU/EEA countries

The control of HIV remains a major public health challenge in Europe. In 2013, 29,157 new HIV cases were reported by 30 EU/EEA countries; corresponding to an overall rate of 5.7 per 100,000 population; 8.9 and 2.6 per 100,000 population for men and women respectively [4]. Migrant populations in the EU/EEA are disproportionately affected by HIV. The categorisation of a migrant case among persons diagnosed with HIV reported to ECDC is based on information on country of birth, country of nationality and/or region of origin.

In 2013, migrants accounted for 35 % of new HIV diagnoses with persons from countries in Sub-Saharan Africa accounting for 15 % of cases and migrants from other regions accounting for 20 % of new diagnoses. Those countries with the highest proportions of new HIV diagnoses among persons originating from outside the country of report in 2013 were Belgium (52 %), Denmark (52 %), United Kingdom (54 %), Ireland (55 %), Norway (63 %), Luxembourg (70 %), Sweden (74 %) and Malta (77 %) [4].

The proportion of new HIV diagnoses among migrants in the EU/EEA has decreased over time, from 44 % of all new diagnoses in 2007 to 35 % in 2012, while absolute numbers of HIV diagnoses among migrants have declined from just over 10,000 in 2007 to about 8700 in 2012 [5]. Over half (53 %) of migrants newly diagnosed with HIV infection during 2007–2012 originated from sub-Saharan Africa. While the proportion of migrants from sub-Saharan African decreased from 59 % in 2007 to 46 % in 2012, the proportion of migrants from other regions increased: cases from Latin America and the Caribbean increased from 14 to 17 % and cases from Central and Eastern Europe increased from 7 to 15 % [4, 6]. Changing patterns of new HIV diagnoses by geographical origin most likely reflect changes in migration flows within and to the EU/EEA, changes in HIV testing as well as changes in incidence in countries of origin [6].

The probable route of HIV acquisition among migrants varies by country or region of origin. Among new diagnoses in migrants from sub-Saharan Africa reported between 2007 and 2012, 87 % were reported as heterosexual transmission and 2 % as mother-to-child-transmission (MTCT). In contrast, 60 % of new diagnoses among migrants from Latin America were among men who have sex with men (MSM) and 33 % due to heterosexual transmission. New diagnoses among migrants from the WHO Region of East Europe were reported as heterosexual (43 %), injecting drug use (23 %) and MSM (16 %) transmission. MSM were the predominant group reported among migrants from Western Europe, East Asia and the Pacific, Australia and New Zealand [6].

While most of the infections in persons born in countries with generalised HIV epidemics were diagnosed for the first time in Europe, it has been largely assumed that most, and particularly those from sub-Saharan Africa acquired HIV in the country of origin. However, there is growing evidence that a significant proportion of migrants have acquired HIV after their arrival in the EU/EEA [7, 8]. For example a UK study found that the proportion of persons with heterosexually acquired HIV who had been born abroad but who acquired their HIV post migration, had increased from 24 % (16–39 %) in 2004 to 46 % (31–50 %) in 2010 (*p* < 0.01) [9]. The aMASE (Advancing Migrant Access to Health Services in Europe) study found evidence that 72 % of migrant MSM probably acquired the virus in their country of residence rather than the one in which they were born [10].

**Fig. 1:**
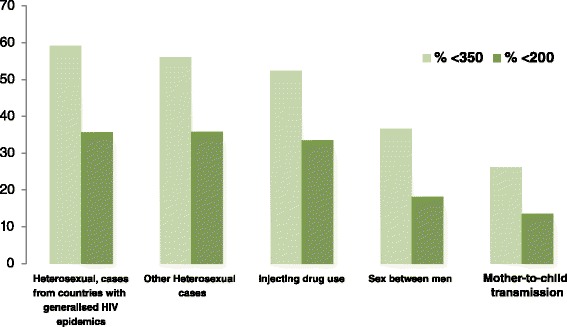
Proportion of HIV cases with CD4 cell count <350/mm^3^ and <200/mm^3^ at diagnosis, by mode of transmission, EU/EEA, 2013 (*n* = 17,526). Source: European Centre for Disease Prevention and Control/WHO Regional Office for Europe. HIV/AIDS surveillance in Europe 2013. Stockholm: ECDC; 2014

Late presentation for HIV diagnosis among migrants is a key concern. In 2013, almost half (47 %) of the 17,529 persons–diagnosed in the 21 EU/EEA countries reporting information on CD4 cell count at diagnosis- were late presenters, defined as a CD4 cell count less than 350 cells/mm^3^ [11]. The proportion of late presenters among migrants originating from sub-Saharan African countries who reported heterosexual transmission was 59 % (Fig. 1).

### HIV and migrant health as political priorities

HIV is a political priority for the EU and the countries of Europe and Central Asia. This is reflected in a number of declarations adopted during the past decade, including the 2004 ‘Dublin Declaration on Partnership to Fight HIV/AIDS in Europe and Central Asia’ and the 2007 ‘Bremen Declaration on Responsibility and Partnership–Together Against HIV/AIDS’. These declarations, and others such as the UNGASS ‘Declaration of Commitment in 2001 and 2006’ and the 2011 Political Declaration on HIV and AIDS, embody the commitment of countries to act on HIV and AIDS and to reach specific targets, including ensuring universal access to HIV prevention, treatment, care and support [12].

The high priority given to HIV is also reflected in European Commission policies and plans, including the European Commission Communication on Combating HIV/AIDS in the European Union and neighbouring countries [13] and the updated Action Plan 2014–2016 [14]. The Communication and Action Plan emphasise the importance of political leadership, involvement of civil society and people living with HIV, human rights, and universal access to services. The Action Plan also highlights the importance of access to prevention and treatment services to all migrants, including undocumented migrants.

In this debate we argue that in the fight against HIV/AIDS in Europe, the provision of antiretroviral treatment (ART) to undocumented migrants should be secured under a global public health approach aimed at both improved health outcomes and reductions in transmission risks. We assess the factors affecting HIV transmission among migrant populations, including undocumented migrants, in Europe. We review national policies in EU/EEA countries, as reported at the occasion of the Monitoring of the Dublin Declaration in March 2014, that restrict access to health care in general and HIV in particular for certain groups of migrants. We outline the rationale for the provision of ART from a human rights and a public health perspective. This discussion is based on literature identified from policy reports from International and European Agencies and the PubMed database using various combinations of search terms appropriate to the HIV epidemiology among migrants, including ‘HIV risk behaviour’, ‘HIV testing’, ‘stigma’, ‘discrimination’, ‘entitlement to HIV care’, ‘socio-economic position’ AND ‘migrants’, along with the terms ‘HIV treatment’, ‘antiretroviral treatment’, ‘viral load’ and ‘community viral load’. We prioritized reports and papers according to their relevance and recency.

## Discussion

### Risk factors affecting HIV transmission among migrants in Europe

HIV incidence in a given population is proportional to the product of the HIV prevalence and the basic reproduction number (R_0_) at that time [15]. The latter describes the number of secondary infections that arise from a primary case. In the equation R_0_ = βcD, term β is the probability of infection per contact, term c is the number of contacts in a given time period and term D the duration of infectivity [16].

Although HIV prevalence is often disproportionally high among some sub-populations of migrants in Europe, prevention interventions cannot act directly on it. The goal of intervention efforts should therefore be to reduce the empirical value of any of the terms of the R_0_, namely the transmission efficiency, contact rate and duration of infectivity [17]. If only one of those terms were zero, HIV would not spread further through the population and the epidemic would be on its way to eradication.

Among available tools to reduce the value of the R_0_ terms, the provision of ART is critical considering its potential to decrease both the transmission efficiency by reducing viral load until undetectable levels (term β) and the duration of infectivity through an early establishment of treatment (term D).

The provision of ART, however, should be an integral part of a combination prevention approach as existing evidence reveals factors at multiple–individual, social and structural–levels affecting the component terms of R_0_ in HIV epidemiology among migrants in Europe, including undocumented migrants. HIV transmission dynamics depend on the respective weight of the component terms of R_0_. All these risk and vulnerability factors act together as in a complex and synergistic interplay, as assumed by infectious disease epidemiology models [17].

Examples of risk and vulnerability factors affecting the component terms of R_0_ are presented in Table 1.

**Table 1:** Risk and vulnerability factors affecting the component terms of R_0_ in HIV epidemiology among migrants

Term	Definition	Factors affecting the term	Intervention to reduce the term’s value
β	Transmission efficiency	Self-perceived risk	Access to HIV information
		Condom availability and use	HIV prevention and risk reduction strategies
		Sexual practices	
		Exposure to sexual violence	
		Stigma	
		Discrimination	
		Disclosure	
		STI co-infections	Access to STI care and treatment services
		Detectable viral load	ART provision
*c*	Contact rate	Number of partners	HIV prevention and risk reduction strategies
		Mixing patterns	
		Size of core group	
*D*	Duration of infectivity	Natural history of infection	Early diagnosis
		Diagnostic interventions	Access to HIV testing services
		Therapeutic interventions	ART provision
		Socio-economic deprivation	Societal interventions

#### Sexual behaviours and attitudes

In populations with high HIV prevalence and high rates of undiagnosed infection, such as in many migrant African communities, assortative sexual mixing may unwittingly increase the probability of HIV exposure [18–20]. A study among individuals who were born in a Sub-Saharan African country and who had migrated to France provided evidence of sexual relations amongst Africans from different countries of origin. This intra-African mixing leads to sexual contacts between migrants coming from low HIV-prevalence and high-prevalence countries and can contribute to the transmission of HIV among African migrants in Europe [21].

One online survey among MSM living in Britain observed that the vast majority of ethnic minority MSM reported unprotected sex with partners from ethnic groups other than their own, particularly with white partners [22]. Central and Eastern European MSM in the United Kingdom were also more likely to have partners from other countries than their heterosexual counterparts. This non-assortative sexual mixing may increase risk of HIV acquisition for migrant MSM moving from low prevalence communities into high prevalence settings such as the London gay scene [23].

Migrants may engage in sexual practices that put them at risk of HIV in their country of residence, but also in their country of origin while visiting family and friends. As such, travellers may act as a bridge population for cross-border transmission of STI including HIV [24–26].

While undocumented migrants are a sub-population of the migrant population in general, structural discrimination against people on the basis of their migration or residence status, and against undocumented migrants in particular puts them at further risk. Exclusion from essential services, protection and justice undermines undocumented migrants’ control over their sexual health. For example, undocumented women are placed at greater risk of enduring sexual violence by factors such as their inability to seek protection and justice from the police without risking arrest, and exclusion from victim’s support and homelessness services. Undocumented women may also practice transactional sex in the context of income and housing insecurity and homelessness, or as sex workers [27, 28]. Further, laws and practices that restrict access to health care services for undocumented migrants can reduce their access to contraception and information on sexual and reproductive health issues, particularly on HIV prevention [28, 29]. A study among a limited population of undocumented migrants–mostly young, Latin-American, single, well-educated and currently working women who accessed services from a community mobile care unit in Geneva, Switzerland–found that women reported multiple sex partners with suboptimal use of STI prevention strategies [30].

#### Uptake of HIV testing services

In EU/EEA countries, a considerable number of individuals remain undiagnosed until they present with an advanced stage of HIV disease [31, 32]. Migrants in Europe experience a disproportionate diagnostic delay and late entry in care (Fig. 1) [4, 33–35]. Many studies have uncovered barriers to HIV testing in the general population as well as in specific sub-populations as for example migrants [36, 37].

At the policy level, barriers concern the actual availability and accessibility of HIV testing services for undocumented migrants [33, 38]. A recent legal analysis showed that only 15 EU Member States entitle them access to HIV testing services [39].

At the provider level, barriers consist primarily of discomfort when approaching the subject of sexual health and HIV, lack of training to increase health care providers’ competence in conducting HIV testing, lack of knowledge about symptoms of undiagnosed HIV infection and logistical barriers such as cost and time constraints [33, 40, 41]. Health care providers and administrators may also be unaware of undocumented migrants’ entitlements to access testing services or face complex procedures for the provision and the reimbursement of these services [38].

At the patient level, barriers include fear of stigma and discrimination–in particular fear of exclusion from one’s own ethnic community–and fear of the disease and the potential for death [42, 43]. Additional barriers are related to risk perceptions with some people not testing due to lack of perceived risk [33, 44–46]. Another barrier to testing is lack of awareness or uncertainty as to where to get an HIV test and undocumented migrants may not be aware that they are entitled to an HIV test [33, 47]. Numerous practical barriers also exist, such as discretionary and inappropriate refusals of care or demands for payment, administrative and financial obstacles [38]. These may have an impact on health seeking behaviour, and the ability to benefit from HIV testing services [47]. Undocumented migrants also express concern that confidentiality will not be respected by medical staff regarding their migration status, and that they may face immigration enforcement as a result of utilising health services [33, 38].

#### Stigma and disclosure

Several studies have highlighted that stigma and discrimination against MSM, migrants, sex workers and against people living with HIV, continues to hamper efforts to prevent new infections and engage people in HIV treatment, care and support programmes [48]. In Europe, there is evidence that HIV-related stigma and discrimination manifests itself within the family, community, workplace, health care system, and within the affected individual. In case of migrants, HIV-related stigma is often exacerbated by marginalisation and restricted entitlements to care [49, 50].

High levels of perceived, anticipated or internalized stigma have been found to yield lower levels of HIV status disclosure; denying the individual the beneficial impact of disclosure in terms of greater social support, the adoption of preventive behaviours and the involvement in HIV care [51, 52].

A qualitative study among HIV positive Black Africans living in the United Kingdom revealed that many feel unable to access community and social support services due to fear of disclosure and related negative consequences [53]. Fear of stigma may deter African and Afro-Caribbean people from disclosing their HIV status to sexual partners [54, 55].

#### Social and economic deprivation

Undocumented migrants are amongst the most impoverished and socially excluded populations in Europe [56]. The mainstream discourse is that irregular migration is an unlawful or even criminal activity which should be controlled and countered. Policy responses increasingly seek to do so by denying undocumented migrants the core elements which constitute a basic standard of living. Their lack of access to adequate housing, education, health care and fair working conditions usually causes poverty and destitution [38]. The poor living and working conditions associated with living in an irregular migration situation, in particular, can have negative impacts on physical and mental health [57–61].

Undocumented migrants mainly seek health care when they are severely ill [38, 62]. The fear of being detected as an irregular migrant, based on the real or perceived exchange of data between health care providers and immigration enforcement, on many occasions prevents and delays migrants from accessing the care they need. Migrants may lack knowledge of the national language, be unfamiliar with the health system, face complicated bureaucracy and be subject to direct and indirect discrimination [38, 63–66].

When necessary health care is denied, delayed or avoided, including HIV testing and treatment, it can prolong periods of poor health and foster chronic health conditions which can lead to further socio-economic exclusion [67].

### National policies regarding the provision of antiretroviral treatment to undocumented migrants

In the EU/EEA, as recorded at the occasion of the monitoring of the implementation of the Dublin Declaration, nearly three quarters of the countries regard migrants as an important sub-population in their national response to HIV [12].

Ensuring access to comprehensive HIV prevention programs as well as to treatment and care services is critical to mitigating the impact of the epidemic [68, 69]. Given that people access treatment and care services through the gateway of HIV testing, international and European guidelines call vigorously for expanding models of HIV testing service delivery. The need to target migrants coming from countries with high HIV prevalence and to provide specific HIV (testing) services was emphasized in the WHO Europe Policy Framework [70] and the ECDC Guidance on HIV Testing [71]. A study of national policies showed that all EU/EEA countries have a set of regulations regarding HIV testing, which create a supportive environment for both client and provider-initiated HIV testing [72]. As of 2010, 15 EU/EEA countries explicitly recommend offering an HIV test to migrants or ethnic minorities [73]. Public health considerations have resulted in about half of EU countries providing free and anonymous HIV testing to undocumented migrants, while access is still restricted in the other countries [74].

**Fig. 2:**
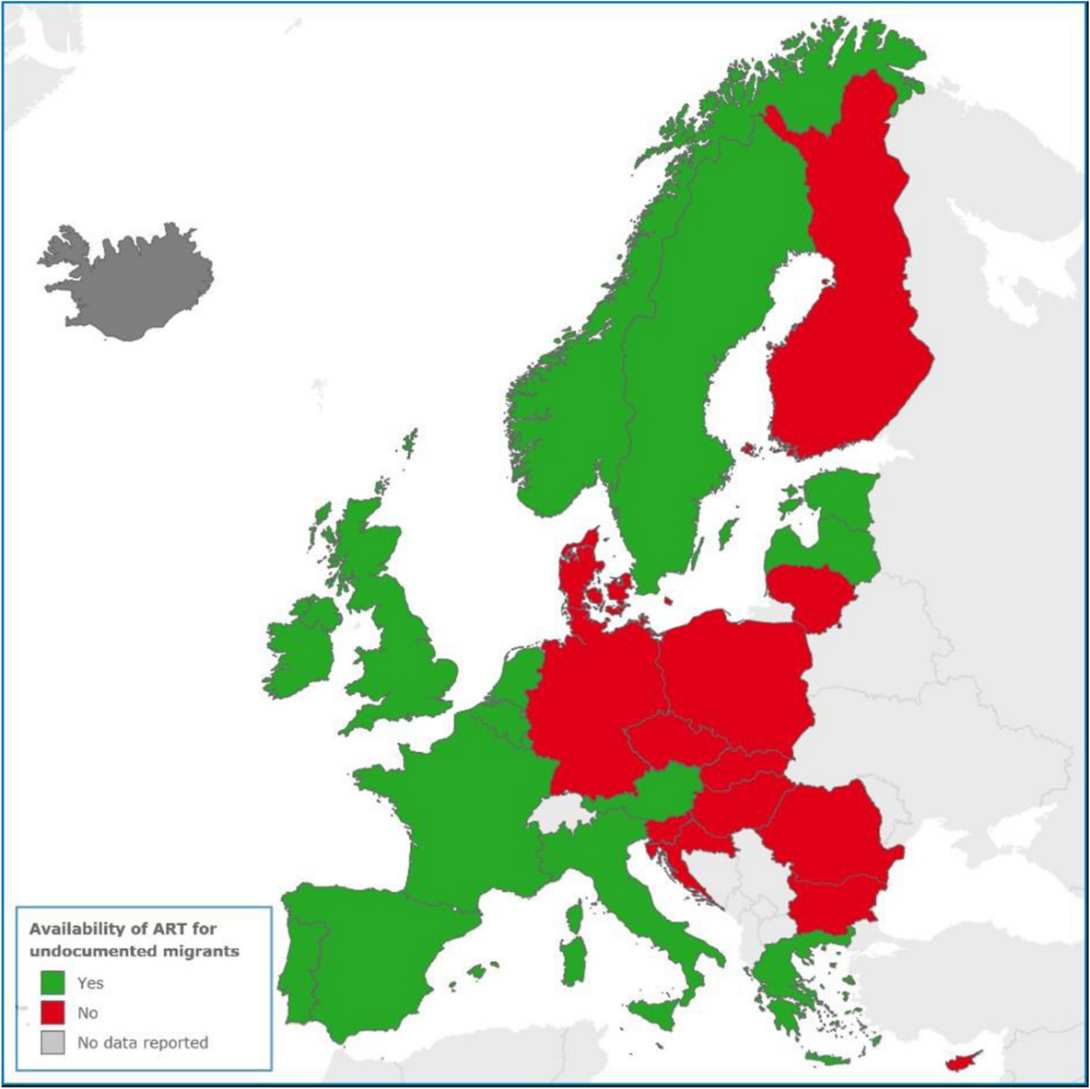
Availability of ART for undocumented migrants in the EU/EEA as reported in March 2014 at the occasion of the monitoring of the Dublin Declaration implementation. Source: European Centre for Disease Prevention and Control. Thematic report: Migrants. Monitoring implementation of the Dublin Declaration on Partnership to Fight HIV/AIDS in Europe and Central Asia: 2014 progress report. Stockholm: ECDC; 2015

In accordance with the basic public health principles of any screening programme, it is essential for all HIV testing programmes to have clear mechanisms to ensure that people who test positive are integrated into HIV treatment and care services [75]. In 2014, national authorities in EU/EEA countries reported that ART was readily available for key affected populations. However, this was not the case for undocumented migrants who face particular challenges in accessing HIV-related services. In 2014, 13 out of 29 EU/EEA countries reported to ECDC that undocumented migrants do not have access to ART treatment (Fig. 2) [76].

In many EU countries, national legislation restricts access to public health care for certain groups of migrants, by linking entitlements to access services to requirements such as residence status, insurance status, and registered employment status [56, 77]. Entitlements to access health care services for undocumented migrants are often regulated by migration legislation, from the perspective that linking access to services to migration status is a component of migration control, rather than a health policy consideration [56]. Further, the economic crisis that has affected many EU/EEA countries has generated austerity measures such as reducing the scope of essential services covered, reducing population coverage and increasing user fees [78, 79]. Obviously, these cuts to public spending on health have an impact on social protection nets, including health care provision [67].

A comparative study of national policies showed that in the majority of EU countries, undocumented migrants are unable to access publicly subsidised health care except in emergencies and even this may be subject to out-of-pocket fees [80]. Some of these provide screening–and a few treatment–for infectious diseases, including HIV, as an additional specialist service. However, the accessibility of such services when there is otherwise no engagement with the health system is very limited [39] In the other EU countries, undocumented migrants are entitled to access further services, ranging from access to primary care to access on a nearly equal footing with nationals. In all countries, entitlement to health care is associated with administrative procedures, which are often complex and bureaucratic, alongside other practical barriers that impair timely access to care [80].

### Lack of access to ART treatment: a human rights violation that counteracts measures to controlling HIV incidence

#### Human right to health care

Since the 1946 Constitution of the World Health Organization (WHO) and the 1948 Universal Declaration of Human Rights (UDHR) the enjoyment of the highest attainable standard of health has been established as a fundamental right of every human being. The human right to health applies universally and was codified into binding law by the International Convention on Economic, Social and Cultural Rights (ICESCR) and the International Covenant on Civil and Political Rights (ICCPR) in 1966.

In 2000, the UN Committee on Economic, Social and Cultural Rights (CESCR) issued “General Comment 14”, an authoritative and binding explanation of Article 12.1 on the right to health of the ICESCR. It states in § 12(b) that governments have legal obligations to ensure that “health facilities, goods and services are accessible to all, especially the most vulnerable of marginalized sections of the population, in law and in fact, without discrimination on any of the prohibited grounds”, defined as “race, colour, sex, language, religion, political or other opinion, national or social origin, property, birth, physical or mental disability, health status (including HIV/AIDS), sexual orientation, civil, political, social or other status”.

In addition, the CESCR specified that States have an obligation to respect the right to health by refraining from denying or limiting equal access for all persons, including asylum seekers and irregular immigrants, to preventive, curative and rehabilitative health services.

All EU countries have ratified the “International Bill of Human Rights” (which is comprised of the UDHR, ICESCR and ICCPR), and thus acknowledge migrants’ equal right to health. Migrants’ access to health care is simultaneously enshrined in other legal instruments. The EU Charter of Fundamental Rights (2000) for example sets out in article 35 that “everyone has the right of access to preventive health care and the right to benefit from medical treatment under the conditions established by national laws and practices”. While this provision is linked to the conditions established in national law, it must be read together with article 21, which prohibits discrimination. Article 11 and 13 of the European Social Charter, on which the provision is based, are also relevant as they guarantee respectively the right to benefit from any measures to enjoy the highest possible standard of health and the right to social and medical assistance for anyone without adequate resources, and the Committee of Social Rights has found this to include undocumented migrants.

A number of policy documents at European level are also relevant. The EU Council Conclusions on Common Values and Principles in European Union Health Systems of 22 June 2006 (C/146) endorsed a joint statement from the Ministers of Health of the EU MS, considering that universality, access to good quality of care, equity and solidarity are the overarching common values and principles, underpinning Europe’s health systems. The EU Council Conclusions on Equity and Health in All Policies of 8 June 2010 urges all Member States to consider policies to “ensure that citizens, and all children, young people and pregnant women in particular, can make full use of their rights of universal access to health care, including health promotion and disease prevention services” [81].

On 8 March 2011, the EU Parliament adopted a resolution ‘Reducing health inequalities in the EU’ (2010/2089 INI), in which it calls on Member States to tackle health inequalities in access to health care for undocumented migrants. The EU Parliament has also adopted the following resolutions which call for improvements in the provision of health care for undocumented migrants: resolution of 4 July 2013 “Impact of economic crisis on access to care for vulnerable groups” (2013/2044 INI) and resolution of 4 February 2014 on undocumented women migrants in the EU (2013/2115 INI). While health is an area of shared competence between the EU and Member States, and health policies are not defined at EU level, these European policy documents reflect political recognition that equity in access to health services for all residents, regardless of status, is necessary both in terms of human rights and health system principles.

Against this background, the European Union Agency for Fundamental Rights recommends that migrants in irregular situation should, as a minimum be entitled by law to access necessary health care services which should not be limited to emergency care only. The same rules for payment of fees and exemption should apply to irregular migrants as to nationals. Finally, the European Union Agency for Fundamental Rights urges EU Member States to disconnect health care from immigration-control policies [56, 82].

#### Treatment and prevention benefits of ART

Restricting access to HIV care not only hampers a state’s compliance with human rights obligations, but it also impacts on the quality of life and survival of HIV infected patients and fuels onward HIV transmission.

The introduction of ART in 1996 was a substantial advance in HIV care. The provision of ART can stop HIV replication on a sustained basis and, as a result, plasma viral load becomes undetectable. Viral suppression allows immune reconstitution to take place, leading to long-term disease remission and prolonged survival. Clinical studies have indicated that maximum benefit in terms of reduced morbidity and mortality is obtained when HIV infection is diagnosed and treated early. Large observational cohort studies such as D:A:D (Data Collection on Adverse Events of Anti-HIV drugs), SMART (The Strategies for Management of Antiretroviral Therapy) and the Collaboration of Observational HIV Epidemiological Research Europe (COHERE) have given convincing evidence that early start and continued treatment with ART not only reduces the classic AIDS defining illnesses, but also non-AIDS defining illnesses linked to HIV such as cardiovascular and renal disease [83–87]. The recent findings from the randomized clinical trial START (Strategic Timing of AntiRetroviral Treatment) have confirmed that earlier ART benefits all HIV-infected individuals [88].

Plasma viral load has also been shown to be a marker of infectiousness. Persons living with HIV with a plasma viral load below the detectable limit are likely to have lower levels of viral load in cervix, rectum, vagina and breast milk. The association between high plasma viral load and high risk of HIV transmission has long been documented [89]. As the highest viral loads are noted immediately after infection, people with acute infection are the most infectious [90]. Observational studies in different populations and mathematical modelling work have demonstrated the secondary benefit of ART in preventing HIV transmission [91].

Reductions in both vertical and heterosexual transmission have been shown to be associated with the receipt of antiretroviral therapy and subsequent reductions in individual viral load [92]. Similarly, reduction in community viral load as result of ART was shown to be a key determinant of decreasing HIV incidence in a cohort of injecting drug users in Vancouver, Canada [93]. In British Colombia, the number of individuals actively receiving ART increased by 54 % between 1996 and 2009 and during the same period, the number of new HIV diagnoses decreased by 52 % [94]. In San Francisco, a decrease in both the mean and total community viral load between 2004 and 2008 was accompanied by decreases in new HIV diagnoses from 798 (2004) to 434 (2008). The mean viral load and total community viral load were significantly associated with new HIV cases [95].

A 92 % reduction in HIV transmission rate was reported in a randomized controlled trial of HIV serodiscordant heterosexual couples in Sub-Saharan Africa in whom the index partner was treated with ART [96]. The real breakthrough for the use of ART as prevention came with the publication of the HPTN052 trial results. HPTN052 was a randomized controlled trial study showing a 96 % reduction in transmission from an infected partner to his or her uninfected stable sexual partner in a heterosexual relationship when the infected partner was put on ART immediately after diagnosis in comparison to couples where the infected partner received treatment only when he or she fulfilled the criteria for initiation of the medication [97]. Similar results have been reported in serodiscordant heterosexual couples in Madrid, Spain [98]. A large observational study, PARTNER, provided preliminary data that support the findings of HTPN052 both for heterosexuals and MSM. The primary aim of PARTNER is to study the HIV transmission risk through condom-less sex if the HIV positive partner is on suppressive ART. So far, there have been no transmissions within couples from a partner with undetectable viral load, in what was estimated as 16,400 occasions of sex for MSM and 14,000 for the heterosexuals [99, 100].

Recognizing the multiple benefits of ART, the WHO consolidated guidelines on the use of antiretroviral drugs for treating and preventing HIV infection (2015) plead for the further scaling up of treatment. These recommendations, based on the findings of the START trial which provided definitive evidence on the benefits of ART treatment, promote expanded eligibility for ART to be started immediately, irrespective of CD4 cell count [101].

To be effective HIV treatment programs should, however, be integrated within a balanced combination prevention framework, including biomedical, behavioural and structural interventions that address the complex interplay of underlying determinants of HIV transmission. Once infected, people should be diagnosed as early as possible after acquiring HIV and they should be offered appropriate prevention and care services and provided the offer of treatment. All this requires an unrestricted access to a continuum of HIV prevention, testing and care services, implemented through a multi-sectorial and participatory approach, recognizing as such the political, economic and social contexts within which all efforts are positioned [102].

The spectrum of engagement in HIV care–also referred to as the HIV treatment cascade–provides a framework for assessing programme implementation and improving programme management so that optimum outcomes can be achieved at each step. The movement towards treatment as prevention has unmasked gaps in the HIV treatment cascade, including late diagnosis, suboptimum linkage to and retention in care, low ART coverage and poor adherence to treatment [103–107]. Leakages from the various steps of the cascade lead to programme inefficiencies and missed opportunities for both treatment and prevention [108, 109].

Inevitably, not offering ART to an affected sub-population makes the cascade leakage worse. Persons who are not linked to care or are poorly engaged in care account for the largest proportion of HIV infected individuals with detectable viral load. These people will consequently continue to unwillingly contribute to the on-going transmission of HIV infection [110, 111]. A current challenge of the HIV epidemic is to reach the highest proportion of overall viral suppression among people living with HIV in order to impact on HIV transmission.

Restricting access to ART treatment for undocumented migrants not only adds a complication to lives that are already constrained and beset by multiple problems, it also constitutes a human rights violation that counteracts measures to control HIV incidence. If Europe is to meet internationally agreed targets by 2020 - 90% of all people living with HIV will know their HIV status, 90 % of all people with diagnosed HIV infection will receive sustained antiretroviral therapy, 90 % of people receiving antiretroviral therapy have suppressed viral loads, addressing the barriers facing undocumented migrants’ access to ART is essential.

## Summary

Migrants represent a significant proportion of HIV cases for all modes of transmission in Europe. Previously it was thought that HIV infections in persons born in countries with generalized HIV epidemics were acquired in the persons’ country or origin. Now, there is growing evidence that a significant proportion of HIV acquisition among migrant populations is occurring after they have migrated to EU/EEA countries.

Migrants are confronted with numerous risk factors that shape patterns of population HIV susceptibility and vulnerability, which simultaneously affect HIV transmission.

Restrictions on access to essential services such as housing, employment, health care, protection and justice for undocumented migrants present additional risks for contracting HIV, through undermining control over sexual health and increasing risk of sexual violence and practices of transactional sex. HIV treatment can only be accessed if infections are diagnosed and persons infected are provided access to treatment services. However, due to numerous legal and practical barriers at policy, provider and patient levels, HIV positive migrants in Europe experience a disproportionate diagnostic delay, and in many cases are unable to access any treatment. Enduring challenging living and working conditions further limit the engagement of undocumented migrants in available health services.

HIV transmission dynamics in migrant populations, including undocumented migrants, depend on the respective weight of all of these risk and vulnerability factors that act together in a multiplicative way. To reduce the incidence of HIV infection, intervention efforts should focus on diminishing the risk and vulnerability factors as being the component terms of R_0_. Within this perspective, the provision of ART to those infected is critical given its potential to decrease both the transmission efficiency and the duration of infectivity.

The use of ART is associated with viral suppression, improved health outcomes and reductions in transmission risks. A current challenge of the HIV epidemic is to reach the highest proportion of overall viral suppression among people living with HIV in order to impact on HIV transmission. People living with HIV who are not linked to HIV care account for the largest proportion of infected individuals with detectable viral load who will continue to unwillingly contribute to the on-going transmission of HIV infection.

The health of migrant communities is linked to that of all EU citizens. Following the recommendations of the European Union Agency for Fundamental Rights, and thereby ensuring access to HIV care for all sub-populations, including undocumented migrants, would not only fulfil the human rights of those populations but also strengthen the control of HIV incidence among those not currently able to access HIV care.
